# Therapeutic and prophylactic effects of oral administration of probiotic *Enterococcus faecium* Smr18 in *Salmonella enterica*-infected mice

**DOI:** 10.1186/s13099-023-00548-x

**Published:** 2023-05-18

**Authors:** Muzamil Rashid, Anmol Narang, Shubham Thakur, Subheet Kumar Jain, Sukhraj Kaur

**Affiliations:** 1 Department of Microbiology, Guru Nanak Dev University, Amritsar, India; 2 Department of Pharmaceutical Sciences, Guru Nanak Dev University, Amritsar, India

**Keywords:** *Salmonella*, *Enterococcus*, Probiotic, Typhoid, Antioxidant enzymes and cytokine

## Abstract

*Salmonella enterica* serotype Typhi causes chronic enteric fever known as typhoid. Prolonged treatment regimen used for the treatment of typhoid and indiscriminate use of antibiotics has led to the emergence of resistant strains of *S. enterica* that has further increased the severity of the disease. Therefore, alternative therapeutic agents are urgently required. In this study, probiotic and enterocin-producing bacteria *Enterococcus faecium* Smr18 was compared for both its prophylactic and therapeutic efficacy in *S. enterica* infection mouse model. *E. faecium* Smr18 possessed high tolerance to bile salts and simulated gastric juice, as treatment for 3 and 2 h resulted in 0.5 and 0.23 log_10_ reduction in the colony forming units, respectively. It exhibited 70% auto aggregation after 24 h of incubation and formed strong biofilms at both pH 5 and 7. Oral administration of *E. faecium* in BALB/c mice infected with *S. enterica* significantly (*p* < 0.05) reduced the mortality of the infected mice and prevented the weight loss in mice. Administration of *E. faecium* prior to infection inhibited the translocation of *S. enterica* to liver and spleen, whereas, its administration post-infection completely cleared the pathogen from the organs within 8 days. Further, in both pre- and post-*E*. *faecium*-treated infected groups, sera levels of liver enzymes were restored back to normal; whereas the levels of creatinine, urea and antioxidant enzymes were significantly (*p* < 0.05) reduced compared to the untreated-infected group. *E. faecium* Smr18 administration significantly increased the sera levels of nitrate by 1.63-fold and 3.22-fold in pre- and post-administration group, respectively. Sera levels of interferon-γ was highest (tenfold) in the untreated-infected group, whereas the levels of interleukin-10 was highest in the post-infection *E. faecium-*treated group thereby indicating the resolution of infection in the probiotic-treated group, plausibly due to the increased production of reactive nitrogen intermediates.

## Introduction

Typhoid is a chronic enteric fever caused primarily due to the infection by *Salmonella enterica* serotype Typhi that is transmitted through contaminated food and water [1]. In 2017, an estimated 14.3 million cases of typhoid were reported worldwide that had the case fatality rate of 0.95% [2]. Typhoid develops into high grade fever in the second week of infection and can persist for more than a month if left untreated [3]. It is an invasive infection that infects many internal organs and, in few cases, cause serious complications such as bradycardia, hepatitis, and acute renal failure [4]. Rapid emergence of multidrug resistance among typhoidal strains of *S. enterica* has increased the severity of the disease and made it difficult to treat [5]. Further, 2–5% of the typhoid patients become chronic carriers of *S. enterica* that is continuously shed in the feces long after the recovery, thereby maintaining the chain of transmission of the infection [6]. Thus, to counter these problems there is a dire need for safe alternative therapeutic agents. One of the alternative options that is being explored is the use of probiotics.

Probiotics are defined as “live microorganisms which when administered in adequate amounts confer a health benefit on the host” [7]. Strains belonging to the genera *Lactobacillus*, *Enterococcus*, *Pedioccoccus*, *Bifidobacteria*, *Saccharomyces* etc. with Generally Regarded As Safe status are used as probiotics for the treatment of various gut-related disorders [8, 9]. Probiotic treatment of infections such as rotavirus [10], and antibiotic-associated diarrhoea [11] have shown promising results in various human clinical trials. Probiotic bacteria inhibit the proliferation of microbial pathogens owing to their ability to produce antimicrobial substances such as hydrogen peroxide, bacteriocins and organic acids [9, 12]. The production of bacteriocins has been considered an important trait in the selection of probiotics as they positively modulate gut microflora [13] and help in better colonisation. Some enterococcal strains such as *E. faecium* SF68, *E. faecium* M74 and *E. faecalis* Symbiflor, are currently being used as probiotics in both humans and farm animals [14–16]. Enterococci are among the first of the few microbial species that colonise the gut of new-born children [17]. As commensals they are present in the gastrointestinal tract [18], mouth and vaginal cavity [19] of humans. Enterococcal probiotics have been tested for their protective effect against human subjects [20, 21].

The use of probiotics for the treatment of typhoid fever is still under exploration phase. Some studies have shown that the oral administration of *Lactobacillus* spp. prior to *S. typhimurium* infection prevented the infection through various mechanisms such as, modulation of host immune response [22], inhibition of *Salmonella*-induced apoptosis of lymphocytes [23] and by increasing the levels of mucin-2, propionic acid in feces [24]. However, the therapeutic effects of probiotic bacteria in in vivo* S. typhi* infection have not been evaluated.

Several enterococcal species have been utilised successfully to preserve processed fruits and vegetables, cheese, dairy products, and meat [25–27]. As part of the starter culture, bacteriocin-producing *Enterococcus* spp. was shown to inhibit food pathogens in cheese [28] and meat [29]. In our previous study [30] we have shown the broad-spectrum antimicrobial effect of enterocin secreted by *E. faecium* Smr18 and its safety in in vitro and in vivo model. Therefore, in this study, we evaluated the probiotic properties of *E. faecium* Smr18 and demonstrated the therapeutic efficacy against *S. typhi* infection in mice. Further, the mechanism of protection were studied by evaluating the antioxidant enzymes, nitric oxide intermediates and modulation of the host immune response.

## Material and methods

### Bacteria

Enterocin-producing *E. faecium* Smr18 [30] used in this study was provided by Dr. Sukhraj Kaur. It was cultured in de Man Rogosa and Sharpe (MRS) broth at 37 °C under stationary conditions. All the chemicals used in the study were purchased from Himedia laboratories pvt. limited Mumbai, India, except where specifically mentioned. *S. enterica* MTCC 733 was procured from Microbial Type Culture Collection (MTCC), Institute of Microbial Technology, Chandigarh, India.

### Gastric and bile juice tolerance assays

Gastric juice tolerance of *E. faecium* Smr18 was evaluated by exposing the cells of *E. faecium* Smr18 to simulated gastric juice (SGJ) made by mixing 3.2 g/L pepsin and 2 g NaCl/L [31]. *E. faecium* cells in their log phase of growth were harvested by centrifugation (9000 g; 10 min at 4 °C) and washed thrice with phosphate buffered saline (PBS; pH 7.2). The cell pellet so obtained was suspended in SGJ at the concentration of 1 × 10^8^ colony forming units (CFU)/mL, and incubated at 37 °C for different time periods. After incubation, the bacterial cells were plated onto MRS agar plates and incubated overnight at 37 °C to check the viability of cells. Bacterial cells suspended in PBS was used as control.

For evaluating bile salt tolerance of *E. faecium*, MRS broth supplemented with 0.3% and 1% (w/v) oxgall were inoculated with 1 × 10^8^ CFU/mL of overnight cultured bacteria in test tubes. The tubes were incubated at 37 °C for different time points. After incubation the bacterial cells were plated onto MRS agar plates for obtaining viable counts [32]. Both the experiments were performed in triplicates.

The effect of phenol on the viability of *E. faecium* was determined by using method of Jena et al. [33]. MRS broth supplemented with 0.4% v/v phenol was inoculated with overnight cultured *E. faecium* cells. After incubation at 37 °C for 8, 16 and 24 h, the culture was serially diluted and spread on MRS agar plates. The cell viability (log_10_ CFU/mL) was calculated by the plate count method.

### Auto aggregation assay

To determine the auto aggregation potential of *E. faecium* Smr18, overnight cultured cells were pelleted down by centrifugation at 9,000 g for 10 min at 4 °C. The cell pellet was washed twice with PBS (pH 7.2) and suspended in PBS. The suspension was incubated at 37 °C for different time points. After incubation period, 1 mL of the suspension from the top of the tube was removed and its absorbance was determined at 595 nm. Auto aggregation percentage was determined by using equation: (1 − At/A0) × 100; Where At is absorbance of suspension at different time points and A0 is absorbance at 0 h. The experiment was performed in triplicates. [34].

### Biofilm formation assay

The ability of *E. faecium* to form biofilm was evaluated by crystal violet assay [35]. Biofilm formation was evaluated in the MRS media at 3 different pH values (3, 5, and 7) and at different time points (24, 48, and 72 h). Cells were cultured overnight in MRS broth and its optical density was set to 0.2 at the wavelength of 590 nm. Microtiter plate having 96 wells was inoculated with 15 µL of culture and 135 µL of MRS broth. The plate was incubated at 37 °C for different time periods to allow the formation of biofilms. After the incubation period, the non-adherent cells were removed by washing the plates three times with PBS. The adherent biofilm was fixed with methanol and stained with crystal violet solution 2% (w/v). After washing off the extra stain, 160 µL of 33% (v/v) glacial acetic acid was used to release the stain from the biofilms, and the absorbance of the biofilms was measured at 595 nm. MRS broth without *E. faecium* was used as control. On the basis of absorbance, the *E. faecium* strain was categorized as, non-biofilm producer if OD ≤ ODC, weak biofilm producer if ODC < OD ≤ 2ODC, moderate biofilm producer = 2ODC < OD ≤ 4ODC, strong biofilm producer = 4ODC < OD, where OD = OD of inoculated well and ODC = OD of control well.

### Determination of antibiotic susceptibility

To determine the antibiotic susceptibility of *E. faecium* isolate to various antibiotics Kirby Bauer method was used [36]. The overnight grown culture of *E. faecium* was spread on MRS agar plate and antibiotic discs were placed onto MRS agar plate with the help of sterile forceps and the plates were incubated overnight at 37 °C. The zone of inhibition was measured in mm and the results obtained were interpreted as per Clinical Laboratory Standards Institute (CLSI).

### Determination of virulence genes

The presence of virulence genes in *E. faecium* Smr18 was determined by polymerase chain reaction (PCR) method. The primers and annealing temperatures for each primer is listed in Table 1 [37, 38]. DNA was isolated from the overnight grown culture of *E. faecium* Smr18 and PCR was performed in 50 µL reaction mixture having 5 µL enterococcal DNA template (50 ng), 25 µL of 2 × PCR master mix, 1 µL of each primer and 19 µL of nuclease free water. DNA was denatured at 95 °C for 4 min followed by 32 cycles of amplification. The amplification was carried out at 72 °C for 1 min. PCR products were analysed on 1.5% agarose gel stained with ethidium bromide and visualized under ultraviolet light using bioimaging system.

**Table 1:** Primer sequences and annealing temperatures for the detection of virulence genes by PCR

Virulence genes	Primer Sequences (5′–3′)	Amplicon (bp)	Annealing temperature (°C)	References
*esp* (enterococcal surface protein)	F: AGATTTCATCTTTGATTCTTGG	510	48	[37]
	R: AATTGATTCTTTAGCATCTGG			
*gel* E (gelatinase)	F: ACCCCGTATCATTGGTTT	419	51	[38]
	R: ACGCATTGCTTTTCCATC			
*cyl* (cytolysin)	F: ACTCGGGGATTGATAGGC	688	58	[37]
	R: GCTGCTAAAGCTGCGCTT			

### Animals and study design

BALB/c mice used in this study were obtained from Central Animal House, Panjab University, Chandigarh. The animal experiments were approved by the Institutional animal ethics committee, Guru Nanak Dev University, Amritsar (Proposal no.226/CPCSEA/2021/32). The animals were housed in polypropylene cages at 25 ± 2 °C temperature under 12 h light/dark cycle at central animal facility Guru Nanak Dev University. All the animals were fed with a standard pellet diet and water ad libitum. Mice were segregated into five groups, with 6 mice in each group. Group G1 (normal healthy control) consisted of vehicle control mice that were orally gavaged with 0.2 mL saline solution. Group G2 is the untreated infection control that were orally infected with live *S*. *enterica* at the dose of 2 × 10^7^ CFUs/mouse suspended in saline solution. Group G3 is the probiotic control that were orally administered with only *E. faecium* Smr18 (10^8^ CFUs/mouse) for 7 days. Mice in the group G4 were orally infected with single dose (2 × 10^7^ CFUs/mouse) of *S. enterica* and after 3 days orally gavaged with *E. faecium* Smr18 (10^8^ CFUs) for 7 days. Mice in the group G5 were orally gavaged with 10^8^ CFUs of *E. faecium* Smr18 for 7 days and on the 8th day infected with single oral dose of *S. enterica* (2 × 10^7^ CFUs/mouse). All the mice were sacrificed at the end of the experiment by cervical dislocation. Blood was collected and sera separated and stored at − 80 °C till further use. The organs, liver and spleen were harvested. A part of the organs was used for estimating the bacterial load and other part was stored at − 80 °C for the estimation of antioxidant enzymes. Bacterial load in the liver and spleen from *Salmonella*-infected groups G2, G4 and G5 was assayed by plating tenfold serial dilutions of tissue homogenates on *Salmonella shigella* (SS) agar plates, the culture plates were incubated at 37 °C for 24 h and CFUs were counted.

### Serum biochemistry

The sera obtained from the blood of mice were subjected to liver and kidney profile analysis with clinical chemistry analyser (Benesphera; model no. c71) by using standard kits (Erba Mannheim, Germany). Liver parameters such as alanine aminotransferase (ALT), aspartate aminotransferase (AST) and alkaline phosphatase (ALP) were expressed in terms of IU/L. Kidney parameters such as urea, uric acid and creatinine were expressed as mg/dl.

### Estimation of antioxidant enzymes

#### Catalase (CAT)

CAT enzyme estimation was carried out according to Bergmeyer and Gawehn, [39] with few modifications. Five percent liver tissue homogenate was prepared in 50 mM potassium phosphate buffer (pH 7). The reaction mixture was prepared by adding 0.05 mL of sample and 2.95 mL of 20 mM hydrogen peroxide. The change in absorbance was measured at 240 nm at 25 °C, and CAT activity was expressed as mM/mg protein according to the formulae: ${\text{b}} = \frac{{\Delta {\text{A}} \times {\text{V}}}}{{\rm{\varepsilon } \times d \times \Delta {\text{t}} \times \rm{v}}}$ × dilution factor; where ΔA = change in absorbance, V = total volume in mL, ε = extinction coefficient, d = path length in cm, Δt = total time for which change was recorded and v is volume of sample.

#### Superoxide dismutase (SOD)

SOD activity in the mice liver homogenates was measured by using the protocol of Kono, [40]. The primary reason for SOD activity is its inhibitory action on the reduction of nitro blue tetrazolium (NBT) dye by superoxide radicals produced by the autooxidation of hydroxylamine hydrochloride. Tissue homogenate (5%) was prepared in 50 mM sodium carbonate buffer (pH 10). The reaction mixture contained 0.250 mL of tissue homogenate, 0.250 mL NBT,0.05 mL triton X-100 and 0.05 mL hydroxyl amine hydrochloride (20 mM) and 0.650 mL of sodium carbonate buffer (50 mM pH 10). The change in absorbance was measured at 560 nm at 30 °C. One unit of SOD is defined as the amount required to inhibit 50% NBT. SOD activity is expressed as:

$$\begin{aligned} & \% {\text{Inhibition}} = \frac{{{\text{Change in absorbance/min}}\,\left( {{\text{blank}}} \right) - {\text{change in absorbance/min}}\,\left( {{\text{test}}} \right) \times 100}}{{{\text{Change in absorbance/min}}\,\left( {{\text{blank}}} \right)}} \\ & {\text{Units/ml}} = \frac{{\% {\text{inhibition}}}}{{50\% \times {\text{V}}}};\,{\text{where V is volume of sample in ml}} \\ & {\text{Enzyme activity}}\,\left( {\text{units/mg protein}} \right) = \frac{{\text{units/ml}}}{{\text{mgprotein/ml}}}. \\ \end{aligned}$$

#### Glutathione reductase (GR)

GR assay was performed by following the methodologies of Carlberg and Mannervik, [41] 5% liver homogenate was prepared in 0.1 mM potassium phosphate buffer (pH7.6). The reaction mixture was prepared by adding 0.1 mL EDTA (3 mM), 0.1 mL NADPH (0.1 mM in 10 mM Hcl pH 7.0) 0.1 mL oxidized glutathione, 0.05 mL sample and 0.650 mL of 0.1 mM potassium phosphate buffer. The change in absorbance was measured at 340 nm at 30 °C for 5 min and the enzyme activity was calculated according to the formula: $b = \frac{{\Delta {\text{A}} \times {\text{V}}}}{{\rm{\varepsilon } \times d \times \Delta {\text{t}} \times \rm{v}}}$ × dilution factor; where, ΔA = change in absorbance V = total volume in mL, ε = extinction coefficient d = path length in cm, Δt = total time for which change was recorded and v is volume of sample.

#### Estimation of sera concentrations of nitrite and nitrate

The pooled sera samples from different groups were filtered and nitrate/nitrite concentration was measured by using nitric oxide estimation kit. Nitrate in the samples was converted to nitrite by adding nitrate reductase enzyme and then nitrite was estimated by adding Griess Reagent 1 (sulfanilamide) and Griess Reagent 2 (N-[1-Naphthyl] ethylenediamine). Finally, the concentration of total nitric oxide (nitrate and nitrite) was calculated by measuring the absorbance of the deep purple azo substance formed at 540 nm. Sample concentrations of total nitric oxide (nitrate + nitrite) and nitrite were derived from the standard curves. The intercept and slope of each standard curve were used to calculate the total nitric oxide or nitrite concentrations of the samples. The concentration of nitrate was calculated by subtracting the nitrite levels from the total nitric oxide levels.

#### Quantification of serum cytokines

Whole blood was obtained by cardiac puncture from all the groups and was collected into tubes containing protease inhibitor. The tubes were then centrifuged at 3400 g for 5 min at 4 °C to separate sera, the sera samples were collected and stored at − 80 °C. For the quantification of cytokines, interferon gamma (IFN-γ), interleukin 10 (IL-10) and transforming growth factor beta 3 (TGF-β3) sandwich ELISA kits (GENLISA, Krishgen Biosystems, India) were used according to the manufacturer’s instructions.

### Statistical analysis

The experiments in this study were carried out in triplicates, the bars on the graph depict mean ± SD. The statistical analysis of the data was carried out using one-way analysis of variance (ANOVA) and Tukey's test and the level of significance was set at 5% (*p* < 0.05). SPSS version 16.0 was used.

## Results

### Probiotic properties

#### Bile salt, gastric juice and phenol tolerance

**Fig. 1:**
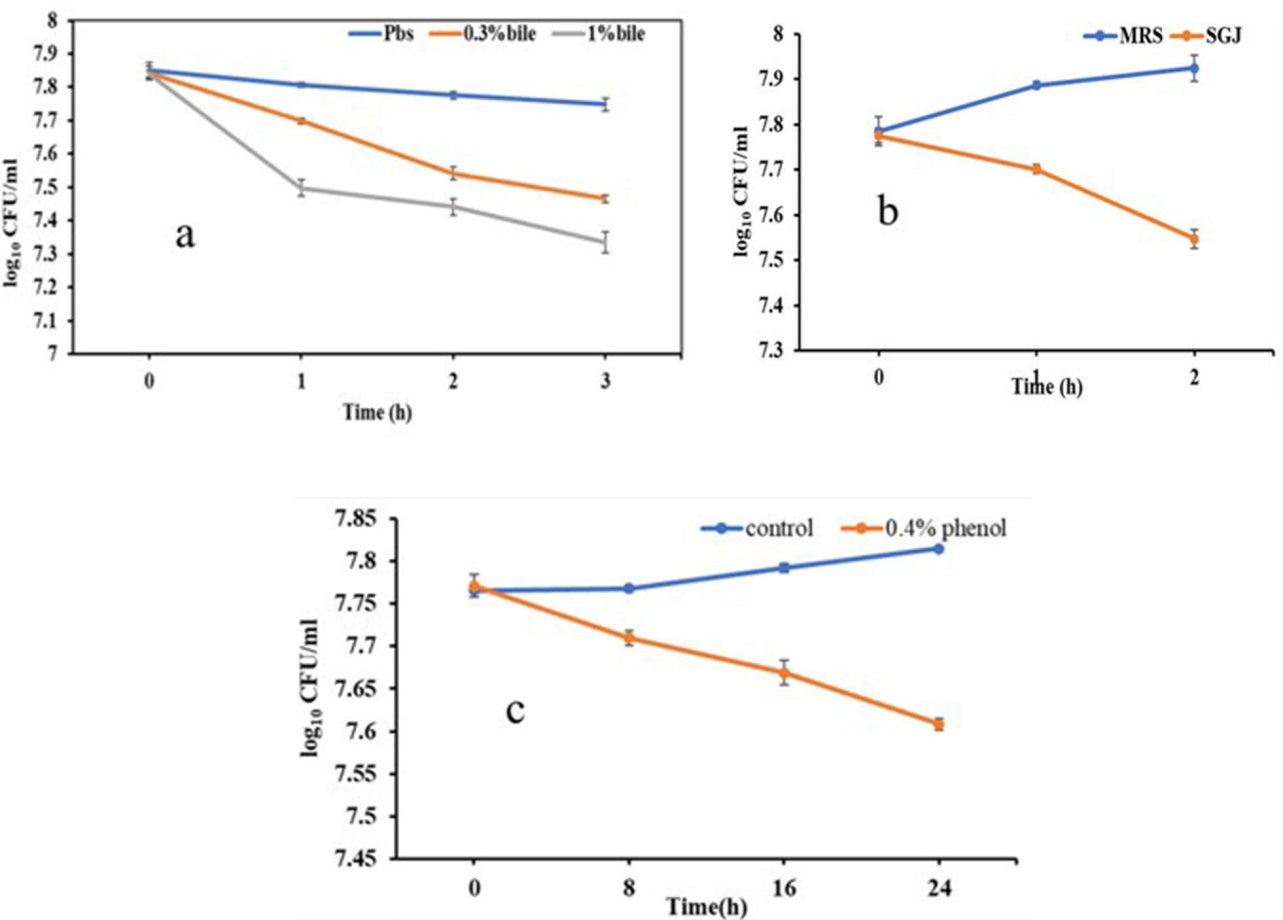
Effect of **a** bile juice and **b** simulated-gastric juice treatment, **c** 0.4% phenol on viability of *E. faecium* Smr18. Error bars are representative of ± SD of the three independent experiments performed in triplicates

Probiotic bacteria needs to survive the harsh conditions in the gastrointestinal tract such as exposure to bile acids, gastric juice, and phenols. Phenols present in the intestine are generated due to the action of microflora that deaminates the aromatic amino acids of the dietary proteins resulting in the formation of phenol, which are inhibitory to the growth of some bacteria. Treatment of *E. faecium* cells with 0.3% and 1% bile salts for 3 h resulted in only 0.4 log_10_ and 0.5 log_10_ CFU reduction, respectively. Further, *E. faecium* appeared to be resistant to the action of SGJ treatment as 2 h exposure of the cells to SGJ resulted in only 0.23 log_10_ CFU reduction (Fig. 1a, b). Further, tolerance of *E. faecium* to phenol was determined. Treatment of *E. faecium* with 0.4% phenol for 24 h resulted in 0.17 log_10_ CFU reduction in viability after 24 h of incubation as compared to MRS control (Fig. 1c).

#### Auto aggregation and biofilm formation

**Fig. 2:**
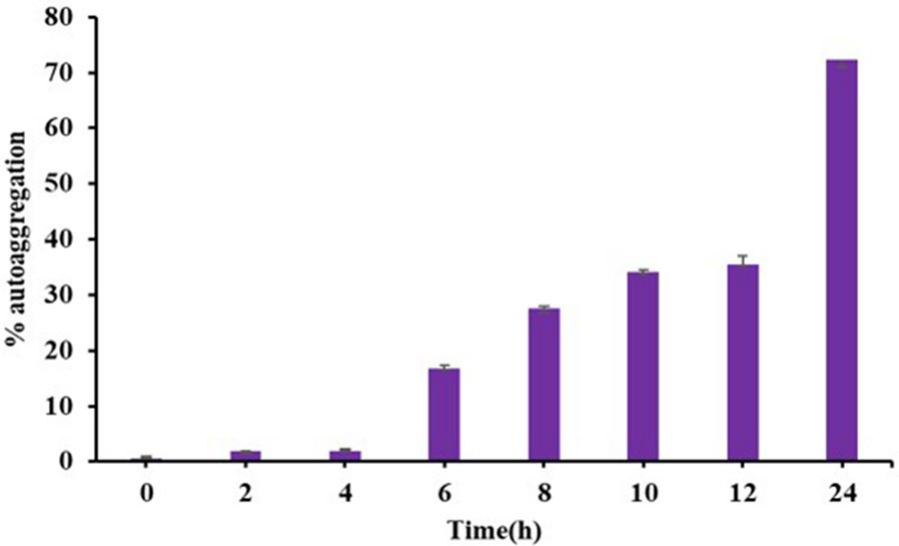
Auto aggregation percentage of *E. faecium*. Error bars are representative of ± SD of the three independent experiments performed in triplicates

Another important property of probiotic strain is its capability to adhere to the host's intestinal epithelium and form biofilm. A correlation between biofilm formation and ability to autoaggregate has been observed in probiotic bacteria. The percentage auto aggregation of *E. faecium* were calculated after different time points that showed that Smr18 exhibited maximum of 70% auto aggregation after 24 h (Fig. 2).

Biofilm formation is an important trait for the persistence of bacteria in vivo. Gut commensals are known to form biofilms in the intestine. As the pH of the gastrointestinal tract varies along its length, therefore formation of biofilm at different pH was observed. Results showed that *E. faecium* formed moderate biofilms after 48 h and strong biofilms after 72 h of incubation at both pH 5 and 7. At pH 3, it formed weak biofilms (Table 2).

**Table 2:** Biofilm-forming potential of the *E. faecium* Smr18

Time	pH 3	pH 5	pH 7
24 h	W	W	W
48 h	W	M	M
72 h	W	S	S

Experiment was performed in triplicates

S, strong biofilm; M, moderate biofilm; W, weak biofilm

Weak biofilm = OD_c_ < OD ≤ 2OD_c_, Moderate biofilm = 2OD_c_ < OD ≤ 4OD_c_, Strong biofilm = 4OD_c_ < OD, OD = OD_600_ of inoculated well, OD_c_ = OD_600_ of uninoculated well

#### Antibiotic susceptibility

The susceptibility of *E. faecium* to various antibiotics was evaluated by Kirby Bauer disk diffusion assay and the zones of inhibition formed were determined. Results showed that the strain Smr18 was susceptible to ampicillin, penicillin-G, ciprofloxacin, linezolid, vancomycin and tetracycline (Table 3) and resistant to the rest of the tested antibiotics.

**Table 3:** Antibiotic susceptibility profile of *E. faecium*

Antibiotic	Concentration (µg/units)	Susceptibility
*β Lactams*		
Ampicillin	10	S^a^
Penicillin-G	10 units	S
*Fluoroquinolone*		
Ciprofloxacin	5	S
Tetracycline	30	S
Linezolid	30	S
*Macrolides*		
Vancomycin	30	S
Erythromycin	15	R
*Lincosamide*		
Lincomycin	15	R
Clindamycin	2	R
Cephalosporin		
Cefuroxime	30	R
Cefotaxime	30	R
Aminoglycosides		
Streptomycin	10	R
Gentamicin	10	R
Amikacin	30	R
Co-trimoxazole	25	R

The experiment was carried out in triplicate

^a^S, susceptible; R, resistant

#### Determination of virulence genes

The PCR of  virulence genes, *esp* (enterococcal surface protein), *gel* E (gelatinase) and *cyl* (cytolysin) by using specific primers showed no amplification for any of the genes in *E. faecium* Smr18, thereby showing it as safe non-virulent strain.

#### Protective efficacy of *E. faecium *in *S. enterica* infection mouse model

Protective efficacy of *E. faecium* was determined against *S. enterica* infection in BALB/c mice. Further, the safety of orally administered *E. faecium* Smr18 was also tested in the mice model. Mortality and body weight of mice in different groups was recorded. Mortality of mice was observed only in the groups G2 and G4. In the group G2, 42% mortality was observed within 10 days of infection with *S. enterica*. One mouse died on day 3 and 2 mice died on day 7 following infection. In G4, 1 out of 6 mice died on day 3 following infection. In all the other groups no mortality was observed.

**Fig. 3:**
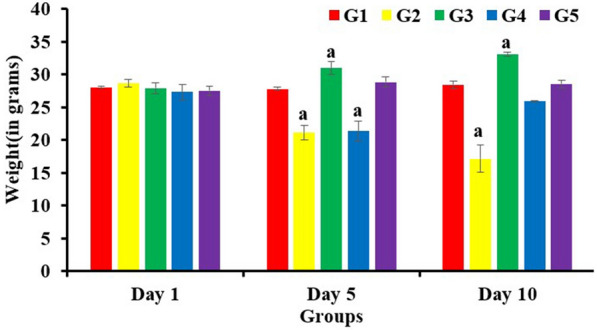
Body weight of animals on day 1, day 5 and day 10 of the experiment. Error bars represent mean ± S.D. a denotes significant (P < 0.05) differences among groups as compared to the respective group on day 1.

Further, the in vivo safety of *E. faecium* smr18 was tested in mice by orally administering high doses (10^8^ CFUs) of viable bacteria for 7 days in the group G3. Oral administration of *E. faecium* for 7 days did not cause any mortality or adverse changes in the behaviour of mice. On the contrary, it resulted in weight gain of mice as shown by significant (*p* < 0.05) increase in the average weight of mice on day 5 and 10 as compared to day 1 (Fig. 3).

On the other hand, in the groups G2 and G4, the average weight of mice showed significant (*p* < 0.05) decrease on day 5 as compared to the weight of normal control animals (G1) on day 1. On day 10, the average weight of mice in G2 further decreased and was reduced by 50% as compared to day 1. Whereas in G4, there was slight gain in the body weight of mice on day 10 as compared to day 5. In the group G5, no change in the average body weights of mice were observed on day 5 and day 10 after the infection (Fig. 3).

The translocation of *S. enterica* to internal organs was determined by evaluating CFU counts in the liver and spleen of all the groups. CFU count of *S. enterica* in the spleen of G2 mice on day 4 and 10 were 5.3 and 6.02 log_10_ CFU, respectively. In liver, the counts of *S. enterica* on day 4 and 10 were 5.23 and 6.60 log_10_ CFU, respectively. *E. faecium*-treated infected groups G4 and G5 showed no growth of *S. enterica* on SS agar plate on the 10th day of experiment (Table 4).

**Table 4:** Viable counts of *S. enterica* in the spleen and liver of mice in various groups

Groups	Log_10_CFU/gm			
	Day 4		Day 10	
	Liver	Spleen	Liver	Spleen
G2	5.230 ± 0.05	5.304 ± 0.01	6.602 ± 0.01	6.021 ± 0.05
G4	5.132 ± 0.07	4.984 ± 0.01	0	0
G5	0	0	0	0

Data are expressed as mean ± SD

#### Biochemical parameters

As infection with *Salmonella* causes invasive disease, it is known to alter the liver and kidney parameters. Therefore, various liver and kidney function tests were performed for all the groups. In the group G2, significant (*p* < 0.05) increase in SGOT, SGPT, ALP, Creatinine, urea, and uric acid levels was observed as compared to the normal mice in group G1 (Table 5). On the other hand, in the *E. faecium*-treated G3 group, all liver and kidney parameters were comparable to the normal control group G1 with no significant changes. Administration of *E. faecium* post-and pre-infection in the groups G4 and G5, respectively resulted in normalised levels of the enzymes SGOT, SGPT and ALP; whereas, a significant (*p* < 0.05) reduction in the levels of creatinine and urea was obtained as compared to the infected control mice in G2. In case of uric acid, a decreasing trend in uric acid levels were observed in both the groups G4 and G5 as compared to G2, although the changes were not significant (*p* < 0.05).

**Table 5:** Biochemical parameters of liver and kidney in different groups of BALB/c mice

Group	Creatinine (mg/dl)	Urea (mg/dl)	U. acid (mg/dl)	SGOT (IU/L)	SGPT (IU/L)	ALP (IU/L)
G1	0.45 ± 0.012^a^	19.92 ± 0.16^b^	4.43 ± 4.43^a^	126.29 ± 6.32^a^	116.26 ± 3.99^a^	120.01 ± 1^ab^
G2	0.64 ± 0.028^c^	36.63 ± 0.63^c^	6.3 ± 6.3^c^	333.91 ± 3.25^b^	288.84 ± 7.82^b^	142.4 ± 2.38^c^
G3	0.47 ± 0.007^a^	18.06 ± 0.33^a^	4.86 ± 4.85^ab^	122.73 ± 5.44^a^	117.73 ± 2.56^a^	128.27 ± 0.64^b^
G4	0.56 ± 0.003^b^	20.19 ± 0.59^b^	5.57 ± 5.57^bc^	118.33 ± 3.06^a^	117 ± 7.54^a^	112.51 ± 2.62^a^
G5	0.57 ± 0.01^b^	17.97 ± 0.05^a^	5.63 ± 5.63^c^	124.67 ± 7.05^a^	112.67 ± 3.55^a^	123.73 ± 8.45^b^

Data are expressed as mean ± SD. Different letters a, b, c, denote significant (*p* < 0.05) differences among the groups

#### Levels of liver antioxidant enzymes

**Fig. 4:**
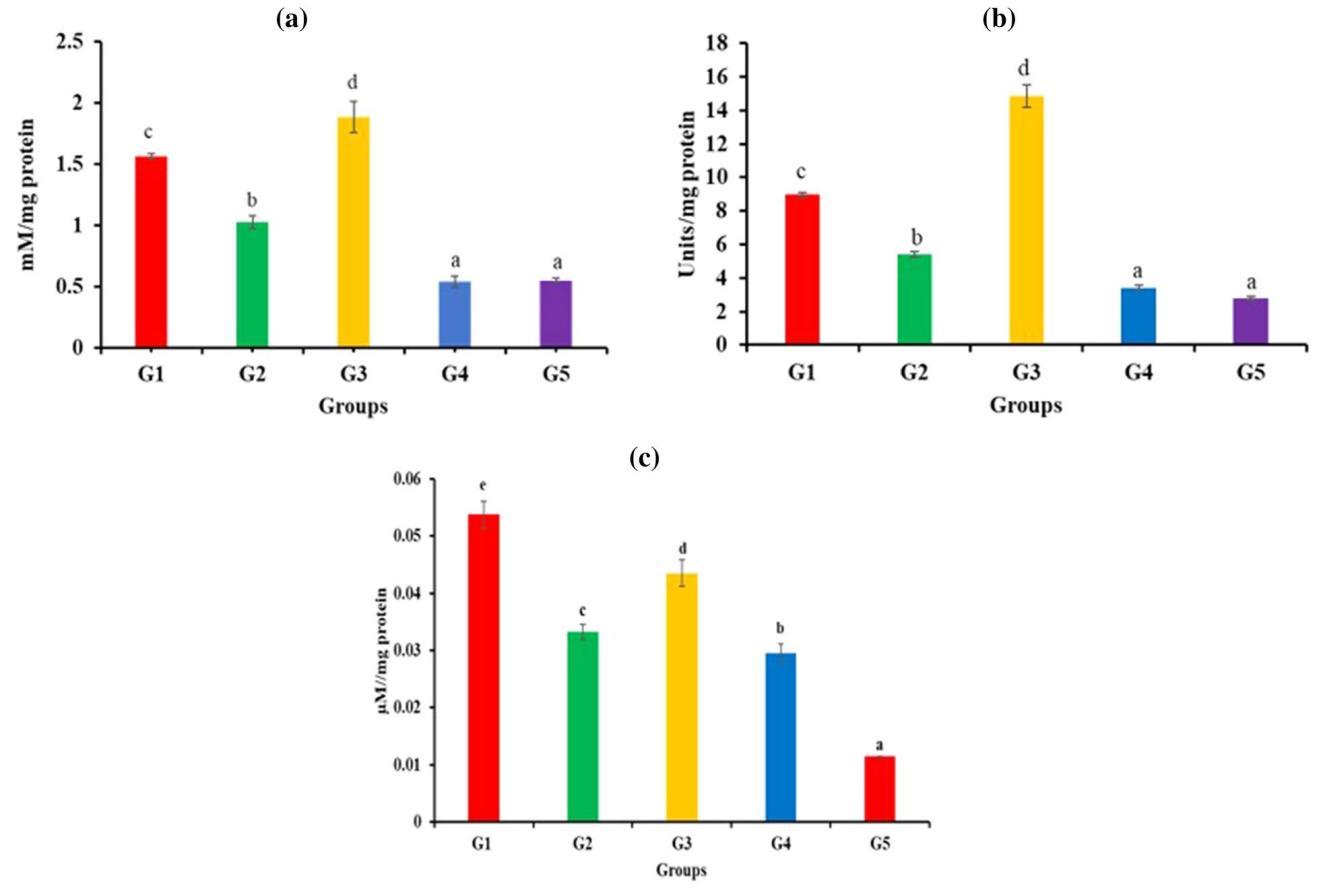
Effect of different treatments on antioxidant activity of liver enzymes, (**a**) catalase, (**b**) superoxide dismutase and (**c**) glutathione reductase in *S. enterica-*infected mice model. Error bars are representative of mean ± SD. Different letters a, b, c, d, and e denote significant (p < 0.05) differences among the groups.

The antioxidant enzymes in the liver of different groups were estimated. *Salmonella*-infection in the group G2 resulted in significant (*p* < 0.05) decrease in the levels of CAT (52%), SOD (65%) and GR (60%) enzymes when compared to normal control (Fig. 4). On the other hand, the levels of both CAT (45%; Fig. 4a) and SOD enzyme (39%; Fig. 4b) significantly increased in the *E. faecium* administered group G3 as compared to the control group G1. The levels of GR decreased (Fig. 4c).

In the *E. faecium*-fed infected groups G4 and G5, the levels of both CAT and SOD enzymes, was significantly (*p* < 0.05) reduced as compared to the infected control (Fig. 4a, b). The levels of GR also showed significant (*p* < 0.05) decrease as compared to the infected groups, but the decrease was more in the group G5 (Fig. 4c).

#### Concentration of nitrite and nitrate in the sera of mice

**Fig. 5:**
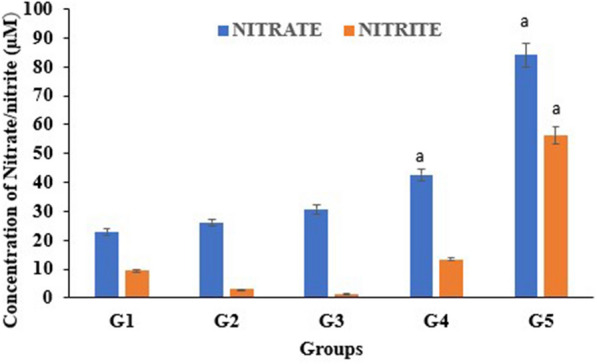
Concentration of nitrites and nitrates in the sera samples of BALB/c mice of different groups. Error bars are representative of mean ± SD. Letter a denotes significant (p < 0.05) difference as compared to the infected control G2.

As the clearance of *Salmonella* from the organs depends on the generation of nitric oxide intermediates, the concentrations of nitrates and nitrites in the sera of mice of different groups were investigated at the end of the experiment. Results showed that the administration of *E. faecium* Smr18 both post-(G4) and pre-infection (G5) significantly (*p* < 0.05) increased the production of nitrate in the sera samples by 1.63-fold and 3.22-fold, respectively as compared to the infected control. On the other hand, the levels of nitrite significantly (*p* < 0.05) increased in the group G5 but not in G4 (Fig. 5).

#### Cytokine levels in the sera of mice

**Fig. 6:**
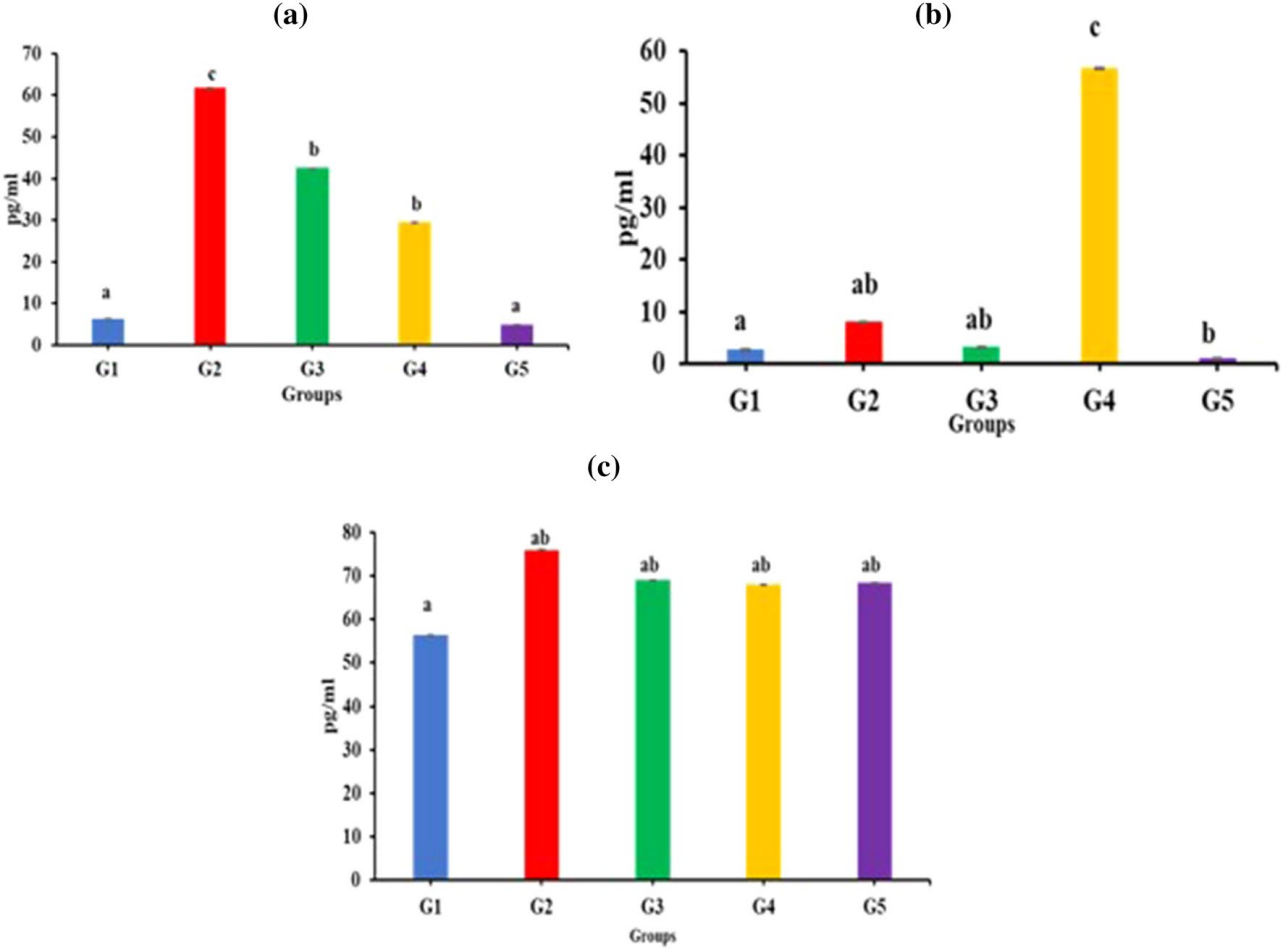
Effect of different treatments on the production of (**a**) IFN-γ, (**b**) IL-10 and (**c**) TGF-β in sera of BALB/c mice. Error bars represent mean ± SD. Different letters a, b, c, denote significant (*p* < 0.05) differences among different groups.

The levels of inflammatory cytokine IFN-γ and anti-inflammatory cytokines IL-10 and TGF-β were estimated in the sera samples of different groups. As shown in Fig. 6a, the levels of IFN-γ were highest in *Salmonella*-infected G2 group (tenfold) followed by G3 (sevenfold). In the group G4 (twofold) and G5 (12-fold), the levels of IFN-γ significantly (*p* < 0.05) decreased as compared to the untreated infected control group G2. The levels of IL-10 showed nonsignificant increase (*p* < 0.05) in both G2 and G3 groups as compared to G1 (Fig. 6b). However, in the G4 group IL-10 levels significantly increased. TGF-β levels were also determined that showed no marked differences in any of the groups (Fig. 6b, c).

## Discussion

In this study, the role of probiotic bacteria *E. faecium* Smr18 in ameliorating *S. typhi* infection in vivo was studied. *E. faecium* Smr18 used in the study is a commensal strain isolated from the healthy human vaginal flora. Strains of *E. faecium* can be commonly isolated from human commensal microflora present in the intestine [42], oral cavity [43], feces and vaginal cavity of healthy humans [44]. Studies have shown that commensal isolates of *E. faecium* are placed in a clade different from that of clinical strains [45] and may not be pathogenic. As part of commensal flora enterococci play important role(s) in enhancing the immunotherapeutic response of cancer drugs [46] down-regulating pro-inflammatory responses in intestinal cells [47], and lowering cholesterol levels in vivo [48].

The probiotic and safety features of *E. faecium* Smr18 was studied as per the ICMR and WHO guideline [49]. Survival under high acidic conditions in stomach and tolerance to bile salts is the most important property for a probiotic strain [50, 51]. Treatment of *E. faecium* Smr18 with bile salts and simulated gastric juice resulted in less than 0.5 log_10_ and 0.23 log_10_ reduction in its cell viability, respectively. Similar tolerance of vaginal commensal *E. faecium* strains to bile salts and gastric juice was reported earlier [44]. Next, the resistance of *E. faecium* to phenol was determined. Aromatic amino acids present in dietary proteins are deaminated by gut bacteria [52], which results in the formation of phenols that is inhibitory to the growth of some bacteria [53]. The maximum concentration of phenol in an adult distal colon range between 0.04–0.05 M [54]. Therefore, resistance of *E. faecium* to 0.4% i.e., 0.05 M phenol was determined. *E. faecium* Smr18 was resistant to phenol as incubation in 0.4% phenol for 24 h resulted in 0.17 log_10_CFU reduction. Similar survival rate of *E. faecium* strains isolated from infant feces and food origin were reported earlier [55].

The ability of probiotic strain to autoaggregate help in biofilm formation in vivo [56, 57]. *E. faecium* Smr18 showed more than 70% aggregation after 24 h of incubation. In another study, *E. faecium* EM485 and *E. faecium* EM925 showed 80 and 78% aggregation, respectively after 24 h incubation [58]. Zommiti et al. [59] reported aggregation ranging between 54 and 96% for different strains of *E. faecium* strains. Further, biofilm formation of *E. faecium* Smr18 was studied that showed moderate and strong biofilm formation after 48 and 72 h of incubation, respectively at both pH 5 and 7.

A growing concern with the use of probiotics in humans is that they may transmit the acquired antibiotic resistance genes to the commensal flora [60]. Secondly, pathogenicity of the strain can be indicated by the antibiotic resistance profile as 80% of the pathogenic *E. faecium* strains are vancomycin-resistant [61]. Therefore, we studied the antibiotic resistance profile of the isolate. Our results showed that Smr18 was quite sensitive to vancomycin, tetracycline, ciprofloxacin, linezolid, and β-lactams i.e., penicillin G and ampicillin. But showed resistance to aminoglycosides, cephalosporin and lincosonamides. Enterococci are intrinsically resistant to these classes of antibiotics [62] and do not pose any threat of transmission of these antibiotic resistance genes. To further verify the safety of *E. faecium* Smr18*,* molecular detection genes of virulence factors gelatinase (gel E), enterococcal surface protein (esp), cytolysin (cyl), present in the pathogenic strains of *Enterococcus* spp. was done. PCR amplification of the virulence genes as per European food safety authority guidelines [63] such as *gel* E, *cyl* and *esp* in the genome of Smr18 by using specific primers yielded negative results, thereby indicating the non-pathogenicity and safety of *E. faecium* Smr18. Similar to our results, virulence genes were not detected in the probiotic strain *E. faecium* SF68 [64].

Further, we studied the safety of Smr18 and its therapeutic efficacy against *S. enterica* in BALB/c mouse model. *Salmonella* strains are known to induce the intestinal cells for their own uptake and once inside the cells they survive, which is an important characteristic of its pathogenicity [65]. The ability of *Salmonella* to survive within the macrophages allow it to be carried to the reticuloendothelial system of different organs [66]. The presence of *Salmonella* in the liver and spleen of the untreated infected mice in the group G2 on 4th and 10th day of infection showed that the pathogen was able to disseminate into the internal organs. Similar dissemination of *S. typhimurium* [67] and *S. enterica* serotype Typhi [68] were reported earlier.

Effort was also made to detect the dissemination of *E. faecium* Smr18 to liver and spleen of mice in groups G3, G4 and G5 by plating the tissue homogenates on *Enterococcus* selective Pfizer agar medium, but no growth was obtained (data not shown), that again indicate the non-pathogenicity of the strain. Further, high mortality and significant (*p* < 0.05) reduction in body weight of mice was observed in the *S. enterica*-infected group G2 at the end of the experiment. Similar weight loss and high lethality was reported by [69] in mice-infected with *S. typhimurium* 3 days post-infection. On the contrary, both pre and post-administration of *E. faecium* Smr18 prevented the weight loss and mortality of mice. Pre-administration of *E. faecium* before *Salmonella* infection completely prevented the dissemination of *Salmonella* to the liver and spleen. Administration in the group G3 resulted in significant increase in body weight of mice on the days 5 and 10. Similar increase in the body weight of broiler chicken fed with probiotic strains of *E. faecium* were reported earlier [70, 71] possibly due to the inhibition of gut pathogens, and maintenance of gut integrity. Thus, administration of *E. faecium* after and before *Salmonella* infection prevented the loss in body weight and the mortality of mice. The results of group G3 show that intake of *E. faecium* Smr18 at high dose was completely safe in mice.

Further, we estimated liver and kidney biomarker enzymes in the sera of mice as *S. enterica* cause invasive infection that results in hepato- and splenomegaly. The increase in the levels of these enzymes in blood is an indication of liver damage as a result of endotoxins, inflammation, or bacterial infection [72]. In our study, *Salmonella* infection in mice (G2 group) caused significant increase (*p* < 0.05) in the sera levels of enzymes such as SGPT (2.48-fold), SGOT (2.64-fold) and ALP (18.6%), creatinine (42%), urea (1.84-fold) and uric acid (42%) as compared to the G1 normal mice, thereby indicating hepatic and renal damage due to *Salmonella* infection. However, in the probiotic-treated infected groups G4 and G5, significant (*p* < 0.05) reduction in the levels of creatinine and urea by 12.5% and 45–50%, respectively was observed as compared to the *Salmonella*-infected group G2. Uric acid levels also decreased by 11.5% but the decrease was not significant in both G4 and G5 groups as compared to G2. *E. faecium* treatment in the groups G4 and G5 normalised the levels of SGOT SGPT and ALP. These results are in accordance with another study, wherein oral administration of probiotic *Bacillus subtills* and *B. coagulans* prior to infection with *S. typhimurium* in rats restored the levels of liver and renal parameters back to normal [73].

Further, our results showed that *Salmonella*-infection caused significant (*p* < 0.05) decrease in the levels of all the 3 tested antioxidant enzymes in liver. These results are consistent with the earlier reports [74, 75]. Reducing the levels of liver antioxidant enzymes is essential for increasing the levels of reactive oxygen species (ROS) and reactive nitrogen species (RNS) that plays important role in controlling infection to *S. enterica* [76]. However, this is not sufficient to clear the pathogen as shown by the presence of *Salmonella* in liver and spleen of the G2 group. Administration of *E. faecium* Smr18, before and after *S. enterica* infection in mice further reduced the levels of antioxidant enzymes leading to enhanced concentration of nitric oxide intermediates that facilitated the complete pathogen clearance from the host. Inducible nitric oxide synthase knock out mice were earlier shown to be extremely sensitive to *Salmonella* infection [77]. Jiang et al. [78] reported increased production of nitric oxide in *Lactobacillus*-treated *Salmonella*-infected macrophages as compared to only *Salmonella*-infected macrophages.

However, the administration of probiotic *E. faecium* Smr18 alone enhanced the levels of antioxidant enzymes, CAT and SOD as shown by other studies [74, 79]. These results indicate that differential antioxidant responses are induced by the host cells in response to pathogenic and probiotic bacteria.

Infection with *Salmonella* is known to cause inflammatory responses in mice as indicated by increase in the levels of IFN-γ. Whereas anti-inflammatory cytokines such as IL-10 and TGF-β are essential for limiting host immune response to pathogens and therefore, is known to get activated at the end after the resolution of infection to restore the normal tissue homeostasis. *S. enterica* infection in mice resulted in significant increase (*p* < 0.05) in IFN-γ levels in the G2 group compared to G1 due to the interaction of the bacteria with the macrophages and dendritic cells that stimulated the production of IFN-γ. However, administration of *E. faecium* after *Salmonella* infection in the group G4 significantly reduced the levels of IFN-γ as compared to G2. Whereas, in the group G5, there was a nonsignificant change in the levels of IFN-γ as compared to normal levels, thereby suggesting that the preadministration of *E. faecium* through inhibiting the dissemination of *S. enterica* prevented the proinflammatory cytokine responses. These results are consistent with another study that showed pre-administration of *L. diolivorans* 1Z to *S. typhimurium*-infected mice resulted in significant decrease in levels of IFN-γ levels [80].

The levels of IL-10 showed nonsignificant changes in the *Salmonella*-infected G2 and probiotic-treated G3 groups as compared to the normal control. IL-10 is an immunoregulatory cytokine produced by the activated T and DC cells to control excessive inflammation leading to the resolution of infection following clearance of the pathogen. The levels of IL-10 showed significant increase (*p* < 0.05) in G4 group as compared to the *Salmonella*-infected G2 group. Another study showed increase in the levels of IL-10 levels following administration of probiotic bacteria *L. casei* in *Salmonella-*infected mice [22]. On the other hand, the levels of IL-10 were significantly lower (*p* < 0.05) in G5 compared to the infected control that again can be explained due to prevention of bacterial translocation to the internal organs in G5. These results are in contrast with previously reported findings wherein, they have shown increased levels of TGβ [24].

## Conclusions

In conclusion, this study shows the potential of probiotic bacteria *E. faecium* Smr18 for the treatment of *S. enterica* infection. Pre-administration of *E. faecium* had prophylactic action against *S. enterica* infection as it prevented the translocation of *Salmonella* to the internal organs shown by the absence of bacteria in the liver and spleen of mice. The mechanism of protective efficacy of *E. faecium* appears to be mediated through causing reduction in the levels of antioxidant enzymes that ultimately leads to enhanced production of nitric oxide intermediates in mice that clear the pathogen from the host.

## Data Availability

The data will be made available without any undue reservation.
